# Bacterial Strategies to Preserve Cell Wall Integrity Against Environmental Threats

**DOI:** 10.3389/fmicb.2018.02064

**Published:** 2018-08-31

**Authors:** Akhilesh K. Yadav, Akbar Espaillat, Felipe Cava

**Affiliations:** Laboratory for Molecular Infection Medicine Sweden, Department of Molecular Biology, Umeå Centre for Microbial Research, Umeå University, Umeå, Sweden

**Keywords:** peptidoglycan, lysozyme, antibiotic resistance, innate immunity, plasticity

## Abstract

Bacterial cells are surrounded by an exoskeleton-like structure, the cell wall, composed primarily of the peptidoglycan (PG) sacculus. This structure is made up of glycan strands cross-linked by short peptides generating a covalent mesh that shapes bacteria and prevents their lysis due to their high internal osmotic pressure. Even though the PG is virtually universal in bacteria, there is a notable degree of diversity in its chemical structure. Modifications in both the sugars and peptides are known to be instrumental for bacteria to cope with diverse environmental challenges. In this review, we summarize and discuss the cell wall strategies to withstand biotic and abiotic environmental insults such as the effect of antibiotics targeting cell wall enzymes, predatory PG hydrolytic proteins, and PG signaling systems. Finally we will discuss the opportunities that species-specific PG variability might open to develop antimicrobial therapies.

## Introduction

The presence of peptidoglycan (PG) as a key component of the bacterial cell wall is one of the defining characteristics of bacteria. PG is an exoskeleton-like macromolecule that envelopes the bacterial cell, preventing them from lysis through osmotic pressure and preserving their shape. PG is composed of β-1,4 linked glycan strands of *N*-acetyl muramic acid (NAM) and *N*-acetyl glucosamine (NAG), cross-linked by short peptide chains. The sugar moieties’ composition is shared by both Gram-negative and Gram-positive bacteria while the nature of their peptides differ between them. In the majority of the analyzed Gram-negative bacteria the basic peptide structure is L-Ala-D-Glu-*meso*DAP-D-Ala-D-Ala while in Gram-positives, the most frequent third amino acid is Lys (Vollmer et al., 2008). Additionally, the cell wall is subjected to numerous changes associated with both the growth cycle and environmental challenges (e.g., antibiotic treatment) (Schneider and Sahl, 2010; Cava and de Pedro, 2014). These changes could occur both in the peptide and/or in the sugar moieties (Vollmer et al., 2008). For instance, PG peptide stems present D-amino acids, which have been suggested to serve as a protection from most of the secreted proteases. However, there are peptidases that can target specifically the muropeptide stems (Uehara and Bernhardt, 2011). Some of these PG-peptidases can target a bacterial cell by different means such as their injection via contact-dependent Type VI secretion system (T6SS). Recent studies have reported the existence of PG modifications, which work as protection mechanisms against these predatory enzymes (Espaillat et al., 2016). Similarly, the sugar moieties are also the target of diverse host secreted antimicrobials such as the lysozyme, a hydrolytic enzyme that cleaves the β-1, 4-glycosydic bond between the NAM and NAG. Some bacteria have devised strategies to overcome host lysozyme-mediated lysis by chemical modification of the NAG and NAM sugars, thereby helping bacteria to evade the host immune system.

**FIGURE 1:**
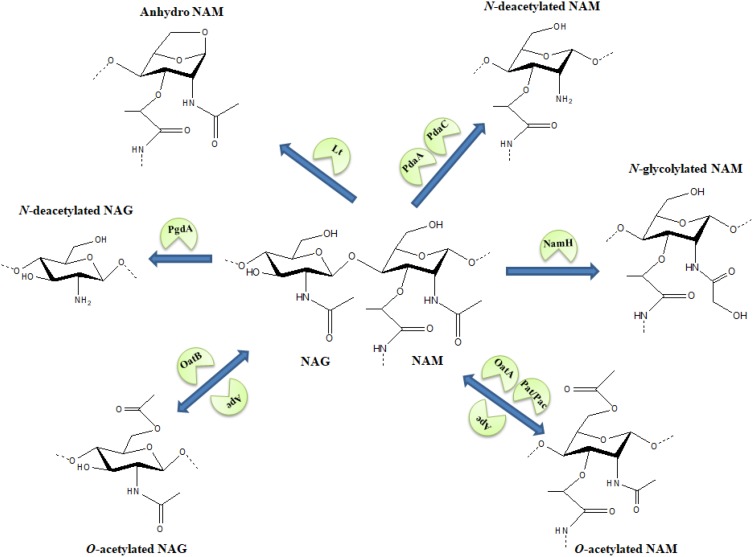
Schematic representation of various modifications in the *N*-acetylmuramic acid (NAM) and *N*-acetylglucosamine (NAG) sugar structures. Arrows carry the responsible enzymes which catalyze the modification. *O*-acetylation of NAG or NAM is a reversible modification where OatA/OatB and Pat/Pac enzymes facilitate the *O*-acetylation of sugars while Ape works as a de-*O*-acetylase for the modification. Lt-lytic transglycosylase.

As cell wall is fundamental for survival, its chemical structure might follow the dynamics proposed by the Red Queen hypothesis effect (Liow et al., 2011), an evolutionary arms race where bacteria would alter the PG chemical structure in order to overcome specific threats to the cell wall. For example, structural variations in the basic moiety of the tracheal cytotoxin (i.e., NAG-anhydro-NAM-tetrapeptide) could lead to a weaker innate immune response (Luker et al., 1995; Knilans et al., 2017). Also, certain pathogens might manipulate the host metabolism as a strategy to evade the immune system and to increase their access to carbon sources (McConville, 2014; Passalacqua et al., 2016). In this review, we summarize PG modifications (**Figure 1** and **Table 1**) that confer protection to diverse antimicrobials, hydrolases and to the innate immune system.

**Table 1:** List of bacterial species comprising various modifications in the sugar moieties of PG.

Modifications	Bacterial species	Reference
*N-*deacetylation of NAG	Gram-negative	
	*Helicobacter pylori*	Wang et al., 2009
	*Shigella flexneri*	Kaoukab-Raji et al., 2012
	Gram-positive	
	*Bacillus anthracis*	Psylinakis et al., 2005
	*Bacillus cereus*	Psylinakis et al., 2005
	*Bacillus subtilis*	Atrih et al., 1999
	*Clostridium difficile*	Peltier et al., 2011
	*Enterococcus faecalis*	Benachour et al., 2012
	*Lactobacillus fermentum*	Logardt and Neujahr, 1975
	*Lactobacillus lactis*	Meyrand et al., 2007
	*Listeria monocytogenes*	Boneca et al., 2007
	*Streptococcus iniae*	Milani et al., 2010
	*Streptococcus pneumoniae*	Vollmer and Tomasz, 2000
	*Streptococcus suis*	Fittipaldi et al., 2008
*N-*deacetylation of NAM	*Bacillus subtilis*	Fukushima et al., 2005
*N-*glycolylation of NAM	*Mycobacterium kansasii*	Azuma et al., 1970
	*Mycobacterium phlei*	Azuma et al., 1970
	*Mycobacterium smegmatis*	Azuma et al., 1970
	*Mycobacterium tuberculosis*	Azuma et al., 1970
*O-*acetylation of NAM	Gram-negative	
	*Agrobacterium tumefaciens*	Weadge et al., 2005
	*Bacteroides fragilis*	Weadge et al., 2005
	*Bacteroides thetaiotamicron*	Weadge et al., 2005
	*Bradyrhizobium japonicum*	Weadge et al., 2005
	*Campylobacter jejuni*	Ha et al., 2016
	*Chromobacterium violaceum*	Weadge et al., 2005
	*Helicobacter pylori*	Wang et al., 2012
	*Moraxella glucidolytica*	Martin et al., 1973
	*Morganella morganii*	Clarke, 1993
	*Neisseria gonorrhoeae*	Dillard and Hackett, 2005
	*Neisseria meningitidis*	Dillard and Hackett, 2005
	*Neisseria perflava*	Martin et al., 1973
	*Photorhabdus luminescens*	Weadge et al., 2005
	*Providencia alcalifaciens*	Clarke, 1993
	*Providencia stuartii*	Clarke, 1993
	*Providencia rettgeri*	Clarke, 1993
	*Providencia heinbachae*	Clarke, 1993
	*Providencia rustigianii*	Clarke, 1993
	*Proteus mirabilis*	Clarke, 1993
	*Proteus myxofaciens*	Clarke, 1993
	*Proteus penneri*	Clarke, 1993
	*Proteus vulgaris*	Clarke, 1993
	*Pseudomonas alcaligenes*	Martin et al., 1973
	Gram-positive	
	*Bacillus anthracis*	Laaberki et al., 2011
	*Bacillus cereus*	Weadge et al., 2005
	*Bacillus subtilis*	Guariglia-Oropeza and Helmann, 2011
	*Enterococcus faecalis*	Pfeffer et al., 2006
	*Enterococcus durans*	Pfeffer et al., 2006
	*Enterococcus faecium*	Pfeffer et al., 2006
	*Enterococcus hirae*	Pfeffer et al., 2006
	*Lactobacillus casei*	Billot-Klein et al., 1997
	*Lactobacillus lactis*	Veiga et al., 2007
	*Lactobacillus plantarum*	Bernard et al., 2012
	*Lactobacillus fermentum*	Logardt and Neujahr, 1975
	*Lactobacillus acidophilus*	Coyette and Ghuysen, 1970
	*Listeria monocytogenes*	Rae et al., 2011
	*Macrococcus caseolyticus*	Bera et al., 2006
	*Micrococcus luteus*	Brumfitt et al., 1958
	*Ruminococcus flavefaciens*	Weadge et al., 2005
	*Staphylococcus aureus*	Bera et al., 2006
	*Staphylococcus epidermidis*	Bera et al., 2006
	*Staphylococcus haemolyticus*	Bera et al., 2006
	*Staphylococcus hyicus*	Bera et al., 2006
	*Staphylococcus lugdunensis*	Bera et al., 2006
	*Staphylococcus saccharolyticus*	Bera et al., 2006
	*Staphylococcus saprophyticus*	Bera et al., 2006
	*Streptococcus pneumonia*	Bonnet et al., 2017
	*Streptococcus faecalis*	Abrams, 1958
*O-*acetylation of NAG	*Lactobacillus plantarum*	Bernard et al., 2012
De-*O-*acetylation	*Campylobacter jejuni*	Ha et al., 2016
	*Neisseria meningitidis*	Veyrier et al., 2013
	*Neisseria gonorrhoeae*	Weadge and Clarke, 2006


## Modifications in Peptidoglycan Sugar Moieties

The sugars present in the glycan backbone of the PG possess the same central chemical skeleton. NAM is the lactic acid ether of NAG and the structural variations in both the sugars are limited to the –NH_2_ group at C-2 and the –OH group at C-6 of the sugars.

### *N*-Deacetylation of NAG

The *N*-deacetylation, removal of the acetyl group at position C-2, from NAG is catalyzed by the enzyme PgdA (Vollmer and Tomasz, 2000). NAG deacetylation is mostly reported in Gram-positive bacteria, e.g., *Bacillus cereus* (Psylinakis et al., 2005), *Enterococcus faecalis* (Benachour et al., 2012), *Clostridium difficile* (Peltier et al., 2011), *Streptococcus suis* and *Streptococcus iniae* (Fittipaldi et al., 2008; Milani et al., 2010). To our knowledge, *Helicobacter pylori* (Wang et al., 2009) and *Shigella flexneri* (Kaoukab-Raji et al., 2012) are the only Gram-negative bacteria having a putative NAG deacetylase homolog.

Mutants in *pgdA* in various bacterial strains are more sensitive to lysozyme and less virulent, thereby NAG deacetylation appears to protect cell wall integrity during infection and also plays a role in evading the host immune system (Vollmer and Tomasz, 2002; Boneca et al., 2007; Wang et al., 2009, 2010; Benachour et al., 2012). In fact, oxidative stress works as environmental trigger for PgdA induction in *H. pylori* (Wang et al., 2009) linking further its function to infection. In *Listeria monocytogenes*, the activity of PgdA is regulated by the cell division proteins GpsB and PBP1A. Deletion of *gpsB* in this bacterium causes an increase in deacetylated muropeptides that leads to a lysozyme resistance phenotype. This phenotype is, however, suppressed upon deletion of PBP1A, thereby underscoring that: (i) besides its PG synthetic activity, PBP1A also serves as an important regulatory partner (Claessen et al., 2008) and, (ii) the relevance of these protein-protein interactions to regulate PgdA cellular activity (Rismondo et al., 2016, 2018). In addition to protecting PG from lysozyme’s action, NAG deacetylation plays also an important role in bacterial predation. *Bdellovibrio* is a bacterium that preys on other Gram-negatives by invading their periplasmic space followed by the release of PG hydrolytic enzymes that digest the prey’s cell wall. During this predator-prey interaction, *Bdellovibrio* deacetylates its own PG to prevent autolysis (Lambert et al., 2016). Similarly, deacetylation also enables coping with the presence of major autolysins in other bacteria, e.g., *Lactobacillus lactis N*-deacetylation decreases the susceptibility of PG to the major autolysin AcmA (Meyrand et al., 2007).

*Helicobacter pylori* PgdA deacetylase activity was suggested *in vitro* using an assay that measured the release of acetic acid from undigested PG (Wang et al., 2009). However, in another study, PgdA failed to deacetylate a variety of possible substrates (e.g., NAG, NAG_3_, etc.) (Shaik et al., 2011) suggesting that further studies will be necessary to identify the actual substrate of this enzyme.

*N*-deacetylation of NAG also helps the bacterium to evade the host immune system. *L. monocytogenes’* deacetylated PG presents a reduced recognition by the NOD1 receptor while PG from the *pgdA* mutant (fully acetylated PG) induces a massive NOD1-dependent IFN-β response (Boneca et al., 2007). In a similar way, a fully *N*-deacetylated *H. pylori* PG (using the *N*-deacetylase from *B. cereus*) completely lost its ability to be sensed *in vitro* by both NOD1 and NOD2 receptors (Boneca et al., 2007), and *Bacillus anthracis* PG containing 88% deacetylated NAG induces very little or no NLRP3 inflammasome activation (Wolf et al., 2016). The reduced recognition of deacetylated PG by NOD1 results also from the inability of lysozyme to degrade it, which limits the availability of NOD1 agonist to the host and thus a weaker innate immune response. While in case of NOD2, the incapability of the receptor to sense the deacetylated PG also contributes to a weaker response (Boneca et al., 2007; Wang et al., 2009; Melnyk et al., 2015). Further details about the innate immune system are in the subsection “Chemical modifications as innate immune modulators.”

### *N*-Deacetylation of NAM

PdaA and PdaC from *Bacillus subtilis* catalyze the removal of the acetyl group from the NAM. *pdaC* deletion mutant shows an increased sensitivity to lysozyme (Kobayashi et al., 2012), while the *pdaA* mutant fails to germinate, given that this activity is implicated in the δ-lactam formation of *B. subtilis* spore cell wall (Fukushima et al., 2002). PdaA is active on denuded PG chains (i.e., PG pre-treated with CwlD, an *N*-acetylmuramoyl-L-alanine amidase, which cleaves the peptide stems) (Gilmore et al., 2004). Although, this activity is important for the spore cortex development in *B. subtilis*, homologs of this enzyme are also encoded in the genome of other non-spore forming microorganisms, e.g., *Rhizobium leguminosarum* (Fukushima et al., 2005). Therefore, the biological implications of PdaA like enzymes in non-sporulating bacteria still need to be determined. One hypothesis is that this activity could help bacteria to evade the innate immune system, as a study on various synthetic structural analogs of muramyl dipeptide (MDP) on NOD2 shows that the acetyl group in NAM is required for binding of MDP to the NOD2 receptor and the activation of the subsequent signaling cascade (Melnyk et al., 2015).

### *N*-Glycolylation of NAM

Four Mycobacterium species namely *Mycobacterium smegmatis*, *M. kansasii*, *M. tuberculosis*, and *M. phlei* present *N*-glycolylated NAM residues, a PG modification catalyzed by NamH (Azuma et al., 1970). In *M. smegmatis, namH* deletion causes increased susceptibility to β-lactam antibiotics and lysozyme (Raymond et al., 2005). Although, *N*-glycolylated NAM confers an enhanced NOD2 recognition, this PG modification seems to have a limited role in *M. tuberculosis* infection (Coulombe et al., 2009; Hansen et al., 2013).

The degree of *N*-glycolylation in the PG varies between species and in response to different antibiotics. *M. tuberculosis* treated with D-cycloserine contains only *N*-glycolyl muramic acid, while similarly treated *M. smegmatis* displays a mixture of *N*-glycolylated and *N*-acetylated PG. Similarly, vancomycin treatment of *M. smegmatis*, consisted of *N*-glycolyl NAM residues only, while in *M. tuberculosis*, this treatment produces a mixture of both the *N*-glycolyl and *N*-acetyl NAM residues (Mahapatra et al., 2005). Blocking PG synthesis at the precursor level (e.g., by vancomycin or D-cycloserine) increases *N*-glycolylation, which is in agreement with NamH being cytoplasmic and acting on the UDP precursor pool.

### *O*-Acetylation of NAM

*N*-acetyl muramic acid *O*-acetylation occurs at the OH group of the C6 of the sugar moiety. Acetylation of NAM seems to be the most widespread PG modification across a great number of Gram-negative and Gram-positive bacteria. Conventionally, the NAM *O*-acetylation in Gram-positive bacteria is catalyzed by the *O*-acetyl transferase OatA (Bera et al., 2005; Rae et al., 2011; Bernard et al., 2012), whereas in Gram-negative bacteria is carried out by a family of enzymes, called Pat or Pac (Dillard and Hackett, 2005; Weadge et al., 2005; Moynihan and Clarke, 2010). Interestingly, *B. anthracis* uses both families of PG *O*-acetyltransferases, i.e., Oat and Pat/Pac. PatA1 and PatA2 is required for separation of *B. anthracis* cells, as well as for proper assembly and attachment of its S-layer (Laaberki et al., 2011). *O*-acetyl transferases Adr and OatA play an important role in cell division of *Streptococcus pneumonia* and *Lactobacillus plantarum*, respectively (Bernard et al., 2012; Bonnet et al., 2017).

*N*-acetyl muramic acid *O*-acetylation confers lysozyme resistance (Bera et al., 2005; Shimada et al., 2010; Bernard et al., 2012) by preventing lysozyme binding to PG due to steric hindrance caused by the bulky acetyl group (Pushkaran et al., 2015). Deletion mutants of *patA* and *patB* in *Campylobacter jejuni* exhibited decreased lysozyme resistance and intracellular survival in macrophage cells (Iwata et al., 2016) while these activities have a minimal impact on *C. jejuni*’s growth and fitness *in vitro* (Ha et al., 2016). In Gram-negative bacteria, *O*-acetylation occurs as a result of the coordinated action of the enzymes PatA and PatB where PatA translocates the acetyl group from the cytoplasm to periplasm and PatB catalyzes the transfer of acetyl groups to NAM (Iwata et al., 2016). Deletion mutant of *patA* in *H. pylori* is susceptible to lysozyme. Moreover, a simultaneous deletion of *patA* and the *N*-deacetylase PgdA makes *H. pylori* five times more sensitive to lysozyme and significantly impaired in intestinal colonization (Wang et al., 2012), highlighting the contribution of both enzymes in *H. pylori* virulence. Importantly, the absence of PatB and Ape1 homologs in *H. pylori* suggests that further studies are needed to validate the role of PatA in the *O*-acetylation of this bacterium. In *Neisseria gonorrhoeae* and *Neisseria meningitidis*, PacA, and PacB are the enzymes required for NAM *O*-acetylation and associated lysozyme resistance (Dillard and Hackett, 2005). Lysozyme sensitivity of *ΔPacA* in *N. gonorrhoeae* is dependent on the loss of two lytic transglycosylases LtgA and LtgD, which compromises the cell wall integrity and permits lysozyme to access the PG (Ragland et al., 2017). In vancomycin-resistant *E. faecalis*, vancomycin treatment increases cell wall *O*-acetylation, which leads to lysozyme resistance and enhanced virulence (Chang et al., 2017)

In some Gram-positive bacteria, e.g., *S. pneumoniae*, *L. monocytogenes*, and *B. anthracis* lysozyme resistance relies on both PG *O*-acetylation and *N*-deacetylation. Only a double mutant in both activities makes the PG susceptible to host lysozyme (Davis et al., 2008; Laaberki et al., 2011; Rae et al., 2011). The activity of OatA in *L. lactis* is regulated by the pyruvate oxidase SpxB, which is induced by the stress-responsive two-component system, CesSR. Induction of OatA by this stress-signaling cascade renders lysozyme tolerance to *L. lactis* (Veiga et al., 2007).

Peptidoglycan *O*-acetylation also contributes to some physiological properties in bacteria other than providing the resistance to lysozyme. In *Staphylococcus aureus*, NAM *O*-acetylation helps to evade the immune system by repressing cytokine production required for differentiation of pro-inflammatory T helper cells (Sanchez et al., 2017). NAM *O*-acetylation also plays a role in septic arthritis. Gonococcal PG induces paw swelling in rats. Notably, *O*-acetylated PG fragments are more arthritogenic compared to non-*O*-acetylated PG (Fleming et al., 1986). These observations are further supported by a study in *S. aureus* strains which shows that the *Δ*OatA mutant in *S. aureus* is less arthritogenic compared to its parental strain (Baranwal et al., 2017).

### *O*-Acetylation of NAG

Contrary to NAM *O*-acetylation, NAG *O*-acetylation is very infrequent in bacteria. To date, only *L. plantarum* has been reported to have *O*-acetylated NAG (Bernard et al., 2011). The *O*-acetylation reaction is catalyzed by the *O*-acetyltransferase OatB. Although *O*-acetylation of NAG plays no role in lysozyme resistance, it inhibits the activity of *N*-acetyl-glucosaminidase Acm2, a major autolysin of *L. plantarum* (Bernard et al., 2011).

### *O*-Deacetylation

As commented above, diverse bacterial species *O*-acetylate their PG to counteract lysozyme’s cell wall hydrolytic activity. Additionally, *O*-acetylation also negatively regulates the activity of endogenous lytic transglycosylases, which require a free hydroxyl group at the C-6 position of NAM. Therefore, to coordinate both lytic and synthetic enzymes, bacteria encode the *O*-acetylesterase Ape for undoing the *O*-acetylation (Weadge et al., 2005). This activity is highly regulated as it only *O*-deacetylates muropeptides with tripeptide stems. Mutants lacking *Ape1* in *C. jejuni* and *N. meningitides* are defective in virulence and have increased chain length and altered cell size (Veyrier et al., 2013; Ha et al., 2016).

As lysozyme’s PG hydrolytic activity is one of the first defense lines employed by the host immune system against bacterial pathogens, most of the above mentioned modifications in the PG glycan backbone that confer lysozyme resistance also improve virulence and/or persistence (Laaberki et al., 2011). For instance, viable but non-culturable (VBNC) *E. faecalis* cells have high levels of PG *O*-acetylation, which inhibits the action of lysozyme (Pfeffer et al., 2006). Interestingly, a study in staphylococci demonstrates that *O*-acetylation of NAM occurs only in the pathogenic strains and not in the non-pathogenic ones (Bera et al., 2006). In this context, it is important to study whether these PG modifying enzymes have emerged during evolution as a mechanism to cope with adverse conditions that a bacterium faces during infection. Further knowledge about the enzyme evolution might unleash the enormous functional diversity of cell wall related enzymes, and the evolutionary processes that gave rise to it.

## Modifications in Peptide Stem

A structural change not only occurs in sugar moieties but also in the peptide stem (Vollmer et al., 2008). This peptide “editing” plays an important role in the fitness and adaptation of bacteria to diverse stress conditions such as those induced by toxic molecules, inter species competition, etc. Some of the chemical modifications and their biological implications are described further.

### Chemical Modifications Providing Antibiotic Resistance

The case of vancomycin resistance is one of the classical examples of a PG modification that provides antibiotic resistance. Vancomycin belongs to the family of the glycopeptide antibiotics. It affects the last step of PG synthesis by binding to the terminal D-Ala-D-Ala of the peptide chain and hence, it inhibits the cross-linking (i.e., transpeptidase) activity of PBPs, which ultimately leads to bacterial death (McGuinness et al., 2017). The resistance was first reported in *E. faecalis* but the same mechanism has been documented on different clinical isolates (Bugg et al., 1991).

Six types of vancomycin resistance have been reported in Enterococci, i.e., VanA, B, C, D, E and G (Courvalin, 2006). The VanA type confers the highest levels of vancomycin resistance (Arthur et al., 1996). This system is encoded by a specific conjugative operon, *VanA*, composed by three elements, (i) a two component system responsible for the detection of vancomycin and the induction of resistance genes; (ii) synthesis of D-Ala-D-Lac dipeptides, catalyzed by a set of genes which convert pyruvate to D-Lac. Then, a cytoplasmic ligase is able to attach D-Ala to the newly synthesized D-Lac; and (iii) a cytoplasmic peptidase that removes the terminal D-Ala-D-Ala of pre-existing peptide stems resulting in an increased pool of modified precursor (terminal D-Lac) over the canonical pool. Altogether, due to this minor change in the PG structure, vancomycin has less affinity to these peptide termini and consequently the bacterium survives in the presence of the antibiotic (McGuinness et al., 2017). The vancomycin resistant type VanC is similar to VanA but it changes the canonical pentapeptide termination from D-Ala-D-Ala to D-Ala-D-Ser, thanks to a transmembrane Ser-racemase. For more detailed information on the molecular basis of the other resistant types see Courvalin (2006).

The PG cross-linking is another structural property of the cell wall that bacteria can modulate to develop resistance to certain antibiotics. PG cross-linking is performed by the transpeptidase activity of high molecular weight PBPs, which uses the energy between the terminal D-Ala-D-Ala bond to cross-link the fourth D-Ala of one peptide stem with the third amino acid (*meso*-DAP or Lys) of an adjacent peptide (Vollmer et al., 2008). Contrary to this canonical DD-cross-link, some bacteria can also display a PG transpeptidase activity that cross-links the cell wall in a different manner. This atypical cross-link is done by a family of enzymes named LD-transpeptidases (Zhao et al., 2017), which connect two *meso* DAP residues from adjacent peptide stems. Indeed, *E. coli* presents 2–7% of its muropeptides cross-linked by LD-transpeptidases (Glauner et al., 1988). Normally, bacteria lacking this type of enzyme do not present any severe phenotype (Sanders and Pavelka, 2013). However, selection of certain mutations on sub-lethal concentrations of Ampicillin can provide high tolerance to different β-lactam antibiotics via a substitution of canonical DD- by non-canonical LD-cross-links. In *E. faecalis*, the mutation occurs in a cytoplasmic DD-carboxypeptidase which changes the muropeptide precursor from penta- to tetra-peptide, favoring the substrate for the LD-transpeptidases (Mainardi et al., 2005). In the case of *E. coli*, it also requires an upregulation of one LD-transpeptidase YcbB, and the activation of the stringent response (Hugonnet et al., 2016). Even though these mutations have been selected under laboratory conditions they provide mechanistic insights about how bacteria can acquire high antibiotic tolerance to β-lactams.

### Chemical Modifications to Combat Bacterial Competition

Bacteria are usually encountered in polymicrobial communities where they establish different types of relations with their co-inhabitants that span from cooperative alliances to fierce competition (Peters et al., 2012). Recently, our group has documented an example of how certain PG modifications could shield the bacterial cell wall during competition (Espaillat et al., 2016). We discovered that the family Acetobacteraceae displays an atypical PG modification: an amidation on the L-center of *meso*DAP. Although this might seem a minor change, it confers a major selective advantage to specific type 6 secretion system (T6SS) driven cell wall effectors (i.e., DD-endopeptidases) that target the D-Ala-*meso*DAP cross-link. When a cross-linked muropeptide presents this modification, the cleavage efficacy of these injected effectors is reduced, suggesting that this modification could be a resistance mechanism to the predatory bacteria. We also found that this bacterial family presented a novel LD-cross-link between the L-Ala on first position of one peptide moiety and the *meso*DAP of an adjacent peptide stem. This LD-cross-linkage also makes the cell wall more tolerant to predatory T6SS endopeptidases (Espaillat et al., 2016). In spite of these *in vitro* data, it remains unclear whether these PG modifications have emerged exclusively as a defensive strategy against surrounding competitors or if they play additional roles in other aspects of the lifestyle of the bacteria.

### Chemical Modifications as Innate Immune Modulators

The innate immune system is the first barrier that eukaryotes display against bacterial infections. In general, the innate immune system is able to recognize pathogen associated molecular patterns (PAMPs) that activate a response which ultimately depends on the molecular activator and the host. Usually, this response is characterized by the production of specific antibacterials (e.g., antimicrobial peptide, AMP) and also, in more complex hosts, the activation of inflammation (Wolf and Underhill, 2018).

One of these PAMPs is the bacterial PG, which is detected by pattern recognition receptors (PRR). Depending on the host, these PRRs could be present either on the surface or in the cytosol of the intestinal epithelial cells (Chaput and Boneca, 2007). The PG can be either actively excreted by bacteria in case of *Bordetella pertussis* and *N. gonorrhoeae* (Wilson et al., 1991; Cloud and Dillard, 2002) or passively due to lysis. Here we give a concise overview of some important aspects on structural diversity in peptide stems and its recognition by the NOD receptors. For more details, there are a number of excellent reviews on PAMP recognition by, e.g., toll-like receptors (TLR), nucleotide-binding oligomerization domain-containing proteins (NOD) and PG recognition proteins (PGRP) (Chaput and Boneca, 2007; Sukhithasri et al., 2013; Wolf and Underhill, 2018).

In mammals, NODs are intracellular proteins which play an important role in PG detection. NOD1 recognizes GM-tri_DAP_ (NAG-NAM-L-Ala-D-Glu-*meso*DAP) as a sensing motif to activate innate immune response. GM-tri_DAP_ being conserved mainly in Gram-negative bacteria makes NOD1 quite specific of sensing these bacteria. NOD2 senses the MDP unit as well as GM-dipeptide, both found in Gram-positive and Gram-negative bacteria (Girardin et al., 2003a,b; Inohara et al., 2003). An *in vitro* study on NOD1 activation in human embryonic kidney cells shows the activation of nuclear factor-kappa B by the presence of *meso*-DAP and *meso*-lanthionine which along with non-sensing of GM-tetrapeptide by human NOD1 strengthens the necessity of an exposed *meso*-DAP in the NOD1 sensing motif (Tohno et al., 2008). Although it could be certainly informative to test the response of these NOD systems to all the possible known variations on the peptide stem, here we want to comment on two modifications on the third amino acid of the peptide stem. The amidation on the L-center of *meso*DAP in the PG of commensal bacteria of Drosophila induces a less potent response of the innate immune system, to not over stimulate the system and to have basal levels of AMP (Espaillat et al., 2016). Moreover, amidation on the D-center of *meso*DAP also produces a weaker NOD1 immune response on human cell lines (Girardin et al., 2003a; Vijayrajratnam et al., 2016). All above mentioned studies about the variations in the peptide stem are directly linked to low sensitivity to the innate immune system, which underlines the necessity of a detailed learning of all possible peptide stem modifications and their implication on the innate immune system.

## Conclusion

With just a few exceptions, PG is a universal component of the bacterial cell wall and thus, a main target of several host produced antimicrobials. Many bacterial pathogens have evolved mechanisms to combat different host defense strategies by modulating their PG structures. Modifications in the PG structure have direct implications on several processes ranging from lysozyme resistance, host immune response and antibiotic resistance. These modifications are important not only as adaptation to specific stresses but also since the cell wall is chemically edited, these modifications will likely have consequences in the activity of other PG-associated enzymes and what will be their physical interactions with these new muropeptides. In this context, the lack of PG editing enzymes would not just make it difficult for bacteria to adapt to stress but also prime a domino effect of PG structural changes with negative consequences in cell wall integrity. This knowledge is valuable for the development of novel antibacterial combinatory therapies to sensitize pathogens that are otherwise non-susceptible to β-lactams. Also, the use of commensal bacteria with specific systems (e.g., T6SS effector against the modification) targeting this PG-editing could be instrumental to devise alternative enzyme-based therapies for the treatment of antibiotic resistant infectious diseases.

A comprehensive study on the PG-modulation strategies which empowers bacterial competition between communities is still in its infancy. Additional chemical analyses of more bacterial PGs is fundamental for gaining a comprehensive understanding of PG variability in nature, as well as under specific conditions (e.g., during infection). The role of bacterial PG persistence and pathogenicity has been a topic of extensive research in recent decades, but still there are many unanswered questions. Continued efforts to understand the cell wall chemical diversity and adaptive enzymatic capacity will surely create new dimensions of antibiotic development strategies.

## Author Contributions

All the authors contributed to the compilation of data and drafted the manuscript.

## Conflict of Interest Statement

The authors declare that the research was conducted in the absence of any commercial or financial relationships that could be construed as a potential conflict of interest.
